# N-carbamylglutamate restores nitric oxide synthesis and attenuates high altitude-induced pulmonary hypertension in Holstein heifers ascended to high altitude

**DOI:** 10.1186/s40104-018-0277-6

**Published:** 2018-09-03

**Authors:** Shuxiang Wang, Arash Azarfar, Yajing Wang, Zhijun Cao, Shengli Li

**Affiliations:** 1 State Key Laboratory of Animal Nutrition, Beijing Engineering Technology Research Center of Raw Milk Quality and Safety Control, College of Animal Science and Technology, China Agricultural University, Beijing, 100193 People’s Republic of China; 2 Faculty of Agriculture, Department of Animal Science, Lorestan University, PO Box 465, Khorramabad, Iran

**Keywords:** High-altitude pulmonary hypertension, Holstein heifers, N-carbamylglutamate, Tibet

## Abstract

**Background:**

High-altitude pulmonary hypertension (HAPH) is a life-threating condition for animals in high altitude, and disturbance of endothelial nitric oxide (NO) synthesis contributes to its pathogenesis. N-carbamylglutamate (NCG), which enhances arginine synthesis, promotes endogenous synthesis of NO. In this study, we determined the effects of NCG on alleviating HAPH in Holstein heifers that ascended to Tibet (Lhasa, 3,658 m).

**Methods:**

Exp. 1, 2,000 Holstein heifers were transported from low elevation (1,027 m) to Lhasa. After being exposed to hypoxia for 1 yr, Holstein heifers were assigned to a healthy group (Control, *n* = 6) with mean pulmonary hypertension (mPAP) < 41 mmHg, and an HAPH affected group (HAPH, *n* = 6) with mPAP > 49 mmHg. Lung tissues were collected to evaluate histopathological changes and the expression of endothelial nitric oxide synthase (eNOS). Exp. 2, ten healthy heifers and 10 HAPH affected heifers were supplemented with NCG (20 g/d per heifer) for 4 wk. Physiological parameters were determined and blood samples were collected on d − 1 and d 28 of the feeding trial.

**Results:**

Expression of eNOS in small pulmonary arteriole intima was higher in the healthy than HAPH group (*P* = 0.006), whereas HAPH group had significantly thicker media and adventitia than healthy group (all *P* < 0.05). The mRNA of *eNOS* and protein level of eNOS were higher in the lungs of heifers in the healthy group than in the HAPH group (both *P* < 0.001), whereas endothelin-1 protein levels were higher in HAPH group than in the healthy group (*P* = 0.025). NCG supplementation decreased mPAP and ammonia (both *P* = 0.001), whereas it increased the expression of eNOS, arginine, and plasma NO (all *P* < 0.05).

**Conclusions:**

The expression of eNOS was decreased in Holstein heifers with HAPH. NCG supplementation decreased mPAP through the restoration of eNOS and endogenous NO synthesis.

**Electronic supplementary material:**

The online version of this article (10.1186/s40104-018-0277-6) contains supplementary material, which is available to authorized users.

## Background

Bovine brisket disease, which occurs in high altitude areas (altitude > 1,524 m) was first defined by Glover and Newsom in 1915, which is initiated by high altitude pulmonary hypertension (HAPH) [1, 2]. Compared with the other mammalian species, cattle exhibit the most severe chronic hypoxic pulmonary hypertension responses [3]. Over 2 million heads of cattle reside at high altitude locations in the United States of America [4], and HAPH affects 3%–25% of some herds transported from low to high altitudes [1]. Acute altitude exposure results in marked reduction of arterial oxygen saturation and oxygen supply to the cardiovascular system [5], whereas it increases mPAP in unadapted individuals. This disease commonly occurs in beef and dairy cattle grown at high altitudes, and there is currently no effective therapy [6–8].

Nitric oxide (NO) is a vasodilator of pulmonary circulation [9], which relaxes vascular smooth muscle tone, and plays a key role in decreasing pulmonary artery resistance and maintaining dilation of the pulmonary vasculature [10]. A reduction in the synthesis of NO by endothelial nitric oxide synthase (eNOS) in the lungs can result in pulmonary vasoconstriction [9], and pulmonary arterial pressure is increased by deleting the *eNOS* gene [11]. Augmented endogenous NO production contributes to suppressing vasoconstriction in Yaks [12]. Furthermore, Tibetans have high NO levels, which confers higher blood flow as a central adaptive mechanism to high-altitude hypoxia [13]. However, eNOS expression and NO production are reduced in rats with pulmonary hypertension [14]. Inhaled NO attenuates pulmonary vasoconstriction [15], and has been shown to improve survival rates in a rat model [16]. However, chronic therapy with inhaled NO has been restricted by its high expense [17]. *L*-arginine, a substrate for eNOS [18], has been used to attenuate pulmonary hypertension in broiler chickens grown at high altitudes [19]. N-carbamylglutamate (NCG), which is a structural analog of N-acetylglutamate [20], promotes urea cycling, and increases the endogenous synthesis of arginine [21], eNOS [22], and NO [23]. Although the effects of *L*-arginine in increasing NO synthesis and eNOS expression, and attenuating pulmonary hypertension have been previously studied [19, 24], the effects of NCG on alleviating HAPH remain unknown.

With the rapid worldwide growth in dairy and beef consumption, large numbers of Holstein and Angus cattle have been moved from low-to high-altitude ranches both in the Qinghai-Tibet plateau of China, and the Rocky Mountains region of the United States of America. However, despite the high incidence rate of HAPH in cattle transported to high altitudes, there is currently no effective treatment available. Therefore, the aim of the current study was to determine 1) pulmonary vascular reconstruction and expression of eNOS in lung tissues of Holstein heifers; 2) effects of NCG on restoring arginine, eNOS and NO synthesis, and alleviating HAPH in Holstein heifers.

## Methods

### Animals and experimental design

The study was conducted in Lhasa, Tibet, China (3,658 m), 2,000 Holstein heifers were transported from Xi’an, Shaanxi Province, China (1,027 m) to Lhasa in 2015 and they were exposed to the hypoxic environment for one year. Diagnosis of HAPH in heifers was based on mean pulmonary artery pressure (mPAP), heifers with mPAP > 49 mmHg were considered to have HAPH, whereas mPAP < 41 mmHg were considered to be healthy heifers [1].

Exp. 1, six healthy Holstein heifers (mPAP: 38.13 ± 1.50 mmHg) with body weight of 557 ± 14 kg, and six HAPH affected Holstein heifers (mPAP: 72.28 ± 1.91 mmHg) with body weight of 480 ± 18 kg, with an average age of 18 ± 2 months were selected. Heifers from each group were euthanized to collect lung tissue for detecting pulmonary vascular reconstruction, and for analyses of mRNA and protein expression of eNOS and endothelin-1 (ET-1).

Exp. 2, ten heifers exhibiting HAPH (460 ± 36.5 kg body weight), and 10 healthy Holstein heifers (497 ± 10.2 kg body weight) with a mean age of 18 ± 2 months, were selected for a feeding trial. The heifers were housed in individual tie-stalls. Heifers were fed three times daily with a total mixed ration (TMR; Additional file 1: Table S1). Physiological parameters were determined, and blood samples were collected after a 30-day adaptation period. Thereafter, heifers were individually supplemented with 20 g of NCG (Asia Pacific Xingmu Technology Co., Ltd., Beijing, China; 98% purity) per day [23] at 07:00 a.m. bytop-dressed feeding onto the total mixed ration (TMR, Additional file 1: Table S1). The physiological and blood parameters were determined after a 4-week feeding period.

### Sample collection

In Exp. 1, heifers were euthanized followed by exsanguination. Lung tissue samples were collected (one dorsally and one ventrally) near the tip of the right lobe. Lung tissue was fixed in 10% neutral buffered formalin and processed for histopathological examination. In Exp. 2, 2 mL of blood was collected from the jugular vein into a 3-mL tube containing 0.25 mL of lithium heparin (1,000 IU/mL). Blood samples were centrifuged at 1,500×*g* for 15 min at 4 °C and plasma was stored at − 20 °C. Samples of the TMR and orts for individual cows were collected daily and pooled weekly during Exp. 2 for analyzing TMR ingredients and chemical composition.

### Physiological parameters measurements

To determine mPAP, the right external jugular vein was pricked using a needle, and a Swan-Ganz catheter (7F) was passed through during heifer awake. A three-way stop cock was used to connect a pressure transducer (Millar Instruments, Houston, TX, USA) to a physiological recorder (Powerlab ML786), and pressure waves were viewed using Chart 5 computer software (AD Instruments, Colorado Springs, CO, USA), as previously described [1]. Measurements were repeated three times and the average of 20 pressure cycles was calculated as the pressure value. Body weights were measured at the beginning of the trial. Peripheral blood pressure and heart rate were measured. Peripheral blood pressure and heart rate were measured using the 9401BP Cardell Veterinary Monitor (Sharn Veterinary, Inc., Tampa, FL), as previously described [25]. Two veterinarians counted the breathing rates at the same time, and measurements were taken from each animal on three consecutive occasions.

### Blood sample analysis

The plasma concentration of eNOS was measured by an enzyme-linked immunosorbnent assay using a primary rabbit polyclonal eNOS antibody (ab5589; Abcam, Cambridge, UK), and a secondary horseradish peroxidase-conjugated goat anti-rabbit antibody (cat. no. 2004; Santa Cruz Biotechnology, CA, USA) as described previously [26]. The intra- and- inter- assay coefficients of variation were 6.0% and 11%, respectively. Plasma NO was measured using a commercial NO kit (Nanjing Jiancheng Bioengineering Research Institute, Nanjing, China) [23]. Plasma urea was measured using a urea analysis kit (ab83362; Abcam, Cambridge, UK), and ammonia was measured using an ammonia analysis kit (ab83362; Abcam, Cambridge, UK). Plasma arginine was determined by fluoro-metric high-performance liquid chromatography methods as described previously [21].

### Histological analysis for pulmonary arteries reconstruction

Lung tissues were sectioned at 8 μm. Verhoeff–Van Gieson staining (VVG, HT25A; Sigma Aldrich, St. Louis, MO, USA) was conducted, and the ratio of staining area to artery area was calculated in both pulmonary arterioles (diameter < 100 μm and diameter ≥ 100 μm) [27]. The media of pulmonary arteries was measured using a primary antibody against smooth muscle-specific alpha-actin (anti α-SMA; Affinity, OH, USA) [28], as described previously [27]. Slides were incubated with a horseradish peroxidase-conjugated goat anti-mouse secondary antibody (sc-2005; Santa Cruz Biotechnology, Santa Cruz, CA, USA) for 30 min at 37 °C. After adding 3, 3′-diaminobenzidine chromogenic, slides were examined and assessed for the ratio of media/(media + lumen + intima). The immunostaining density of eNOS in the endothelium was measured using an eNOS antibody (ab5589; Abcam, Cambridge, UK) [26], following the manufacturer’s protocol [14]. Six slides (five arteriole within each section) were selected for taking pictures using Leica microscope (DM2500, Leica Camera AG, Solms, German) and quantification using Image-Pro Plus software (Media Cybernetics, Rockville, MD, USA).

### Reverse-transcription polymerase chain reaction (RT-PCR) for eNOS and ET-1 in lung tissues

Total RNA was isolated using Trizol (Invitrogen, Carlsbad, CA, USA), and 2 μg of DNAse-treated total RNA was reverse transcribed using a reverse transcriptase kit (Promega, Madison, WI, USA). Reverse-transcription polymerase chain reaction was performed using Biosystems 7300 System (Foster, CA, USA) with a master mix (4367659; SYBR Green; Foster, CA, USA). The primer pairs used for endothelin-1 (*ET-1)*, *eNOS* and *β-actin* amplification and the reaction conditions were as follows as described previously [29, 30]. The reaction efficiency for each primer assay was (*ET-1 =* 94.54%, *eNOS* = 96.84%, *β-actin* = 98.44%, respectively). Data obtained from RT-PCR were normalized against *β-actin*. The relative quantification of gene amplification by RT-PCR was performed using cycle threshold (Ct) values. The comparative Ct value method was employed to quantify expression levels for *eNOS* and *ET-1*, as described previously [22].

### Western blot for eNOS and ET-1 in lung tissues

Total protein (100 mg) was extracted using a protein extraction kit (Beyotime, Beijing, China). Protein concentrations were measured using a bicinchoninic protein assay kit (CoWin Biotech, Beijing, China). Protein extracts were resolved on 5.0% SDS polyacrylamide gels. Proteins were transferred onto a polyvinylidene fluoride membrane (Millipore, Temecula, CA, USA), and incubated with 5% bovine serum albumin (BSA) at 4 °C overnight. The membranes were subsequently incubated with primary ET-1 antibody (ab2786, Abcam, Cambridge, UK, 1:500 dilution), eNOS antibody (ab5589, Abcam, Cambridge, UK, 1:1,000 dilution) [26] or GAPDH antibody (Sigma-Aldrich, St. Louis, MO, USA, 1:10,000 dilution). After incubation, the membrane was washed three times with phosphate buffer solution containing tween (1 × phosphate buffer solution + 0.01%Tween-20). A horseradish peroxidase conjugated goat anti mouse secondary antibody (Jackson, PA, USA) was applied for ET-1 (1:10,000 dilution);horseradish peroxidase-conjugated goat anti rabbit secondary antibody (1:10,000 dilution) was used for eNOS; horseradish peroxidase-conjugated rabbit anti mouse secondary antibody (1:10,000 dilution) was used for GAPDH (G9545, Sigma-Aldrich, St. Louis, MO, USA,). Gel images were scanned using a Gel Image system (ver.4.00; Tanon, Shanghai, China). Target bands were quantified by densitometry using Image-Pro Plus (Media Cybernetics, Rockville, MD, USA).

### TMR ingredients and chemical composition analyses

All TMR and orts samples were dried at 65 °C in a forced-air oven (Model 2000; Experimental Mill, China) for 48 h to a constant weight, ground through a 1-mm screen using a Wiley mill (standard model 4; Arthur H. Thomas Co., Philadelphia, PA), and analyzed for dry matter (DM), crude protein (CP; method 4.2.08; AOAC 1990), ether extract (method 920.85; AOAC 1990), ash (942.05; AOAC 1990), calcium and phosphorus (method 945.46; AOAC 1990), acid detergent fiber (ADF; expressed exclusive ash) (method 973.18; AOAC 1990) [31], and neutral detergent fiber (NDF) [32]. The NDF was determined using sodium sulfite without α-amylase and was expressed exclusive to ash using the Ankom 200 fiber Analyzer (Ankom Technology, Fairport, NY, USA). The chemical composition of the TMR is presented in Additional file 1: Table S1.

### Data analyses

Data are expressed as means ± SEM or pooled SEM. Statistical comparisons were performed using unpaired Student’s *t*-test. The effects of NCG were analyzed using two-way repeated-measures ANOVA, with Bonferroni post hoc correction for significant interactions, NCG treatment and Holstein heifers’ state as main factors. Differences among individual treatment within a column were tested by Duncan’s multiple comparison when a significant interaction between the main effects was observed. Statistical analyses were performed using Graph Pad software (Graph Pad Software, Inc., La Jolla, CA). Statistical significance was declared at *P* ≤ 0.05.

## Results

### Differences in pulmonary vascular reconstruction

**Fig. 1:**
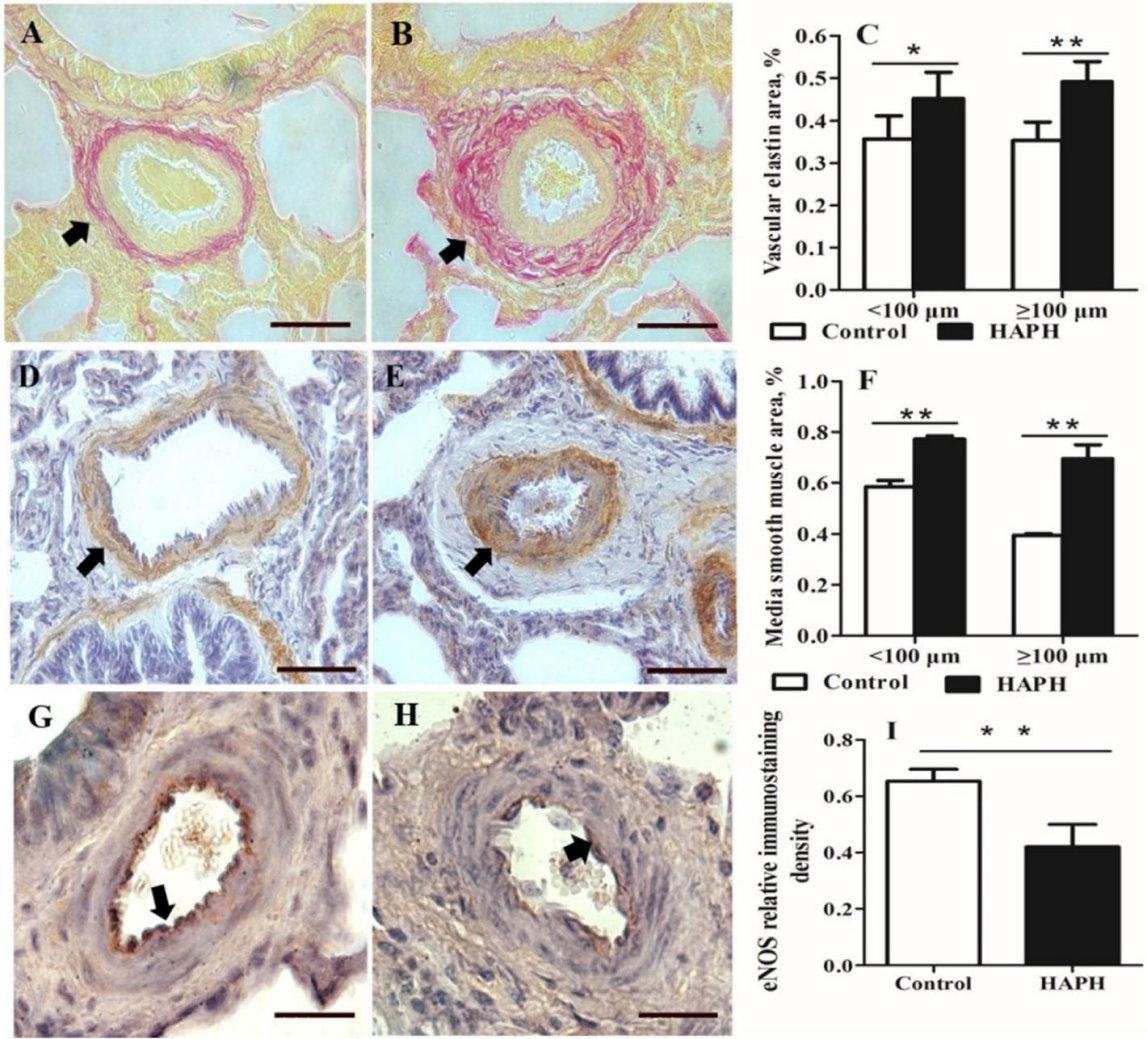
Immunohistochemical staining of small pulmonary arteries in lung tissue sections. (Control = healthy Holstein heifers, HAPH = Holstein heifers with high altitude pulmonary hypertension. Means ± SEM (*n* = 6), *P < 0.05; **P < 0.01). (**a**) Verhoeff–Van Gieson staining (VVG) for elastin in small pulmonary arteries from healthy heifers. (**b**) Representative images of lung histological sections of heifers in HAPH group. Arrows indicate the proportional volume of elastin-positive adventitia. (**c**) Bar graphs representing the percentage area of vascular elastin in the small (diameter < 100 μm) and other (diameter ≥ 100 μm) pulmonary arteries. (**d**) Immunohistochemical staining of alpha smooth muscle actin (α-SMA) in small pulmonary arteries of Healthy heifer. (**e**) Representative images of histological lung sections from Holstein heifers with HAPH. Arrows indicate the proportional volume of the media layer. (**f**) Bar graph representing the percentage area of vascular media in small (diameter < 100 μm) and other (diameter ≥ 100 μm) pulmonary arteries. (**g**) Immunohistochemical staining of endothelial nitric oxide synthases (eNOS) in the healthy heifers. (**h**) Representative images of lung histological sections from heifers in the HAPH group. Arrows indicate immunostaining of eNOS. Scale bar = 50 μm. (**i**) Bar graph representing the relative immunostaining density of eNOS in pulmonary arterial endothelium (75 μm ≤ diameter < 100 μm). Scale bars = 100 μm

The adventitia of pulmonary arteries were significantly thicker in the HAPH group compared with healthy group; [(diameter < 100 μm; *P* = 0.033) and (diameter ≥ 100 μm; *P* = 0.001)] (Fig. 1a-c). A significantly higher percentage of vascular media area was observed in the HAPH group compared with the healthy group [(diameter < 100 μm; *P* = 0.006) and (diameter ≥ 100 μm; *P* < 0.001)] (Fig. 1d-f). Immunostaining densities of eNOS were significantly lower (*P* = 0.006; Fig. 1g-i) in the HAPH group than in the healthy group. Moreover, the intima of pulmonary arteries in the healthy group were significantly thicker compared with the HAPH group (Fig. 1g-h).

### Expression of eNOS and ET-1 in healthy and HAPH affected heifers

**Fig. 2:**
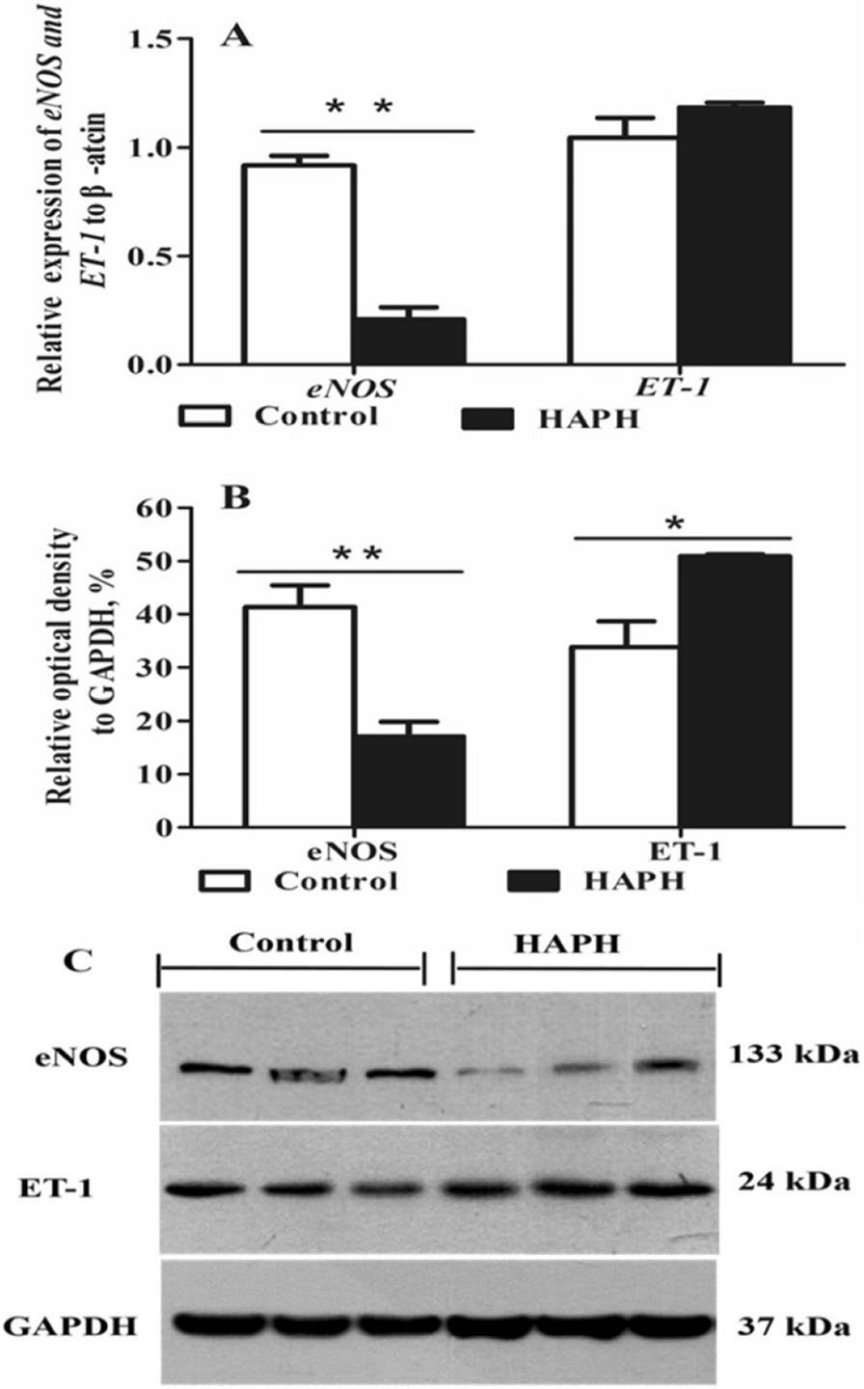
Expression of endotheline-1 and endothelial nitric oxide synthases mRNA and protein in lung tissues. Control = healthy Holstein heifers, HAPH = Holstein heifers with high altitude pulmonary hypertension. Means ± SEM (*n* = 3), *P < 0.05; **P < 0.01. Relative mRNA levels of endotheline-1 (*ET-1*) and endothelial nitric oxide synthases (*eNOS*) in lung tissues. The expression of *β-actin* served as an internal control. Bar graph representing the relative ET-1 and eNOS protein levels. Glyceraldehyde 3-phosphate dehydrogenase (GAPDH) protein expression served as an internal control. Western blot showing ET-1 and eNOS protein levels in lung tissues

In lung tissues, *eNOS* mRNA and protein levels of eNOS were higher in the healthy group than in the HAPH group (*P* < 0.001 and *P* = 0.008; Fig. 2a and b, respectively). Furthermore, HAPH group exhibited higher ET-1 protein levels compared with the healthy group (*P* = 0.025; Fig. 2c).

### Effect of NCG supplementation on physiological parameters

Heifers in the HAPH group had greater mPAP than heifers in the healthy group (*P* = 0.001; Table 1). Supplementation of NCG significantly reduced mPAP in both groups (*P* = 0.001). Supplementation of NCG had a tendency to decrease systolic blood pressure (*P* = 0.068), and a tendency to increase breath rate (*P* = 0.065) in HAPH group. For systolic blood pressure the state × treatment interaction was significant (*P* = 0.005) and supplementation with NCG significantly reduced systolic blood pressure only in the HAPH group.

**Table 1:** Effects of supplementation with N-carbamylglutamate (NCG 20 g/ d per heifer) on physiological parameters^a^

Item^b^	mPAP, mmHg	Breathing, times/min	Systolic, mmHg	Diastolic, mmHg	Mean, mmHg	Heart rate, beats/min
Healthy before^c^	37.84	34.23	104.11^B^	53.86	71.18	83.09
Healthy after^d^	33.60	34.59	105.59^B^	55.55	73.62	82.73
HAPH before	72.60	27.97	111.63^A^	58.62	75.72	86.37
HAPH after	67.13	30.10	104.85^B^	55.95	73.30	82.48
SEM	3.111	1.028	1.003	0.958	1.018	1.864
Main effects						
Status						
Healthy	35.72	34.41	104.94	54.71	72.40	82.91
HAPH	69.87	29.04	108.24	57.29	74.51	84.43
Treatment						
Before	55.22	31.10	107.87	56.24	73.45	84.73
After	50.37	32.35	105.31	55.75	73.46	82.61
*P*-value						
State	0.001	0.076	0.238	0.278	0.400	0.747
Treatment	0.001	0.065	0.068	0.733	0.996	0.486
State×Treatment	0.087	0.136	0.005	0.139	0.138	0.561

^A,B,^ Within a column, means without a common superscript letter differ (*P* < 0.05)

^a^Healthy = healthy Holstein heifers, HAPH = Holstein heifers with high altitude pulmonary hypertension. Means and pooled SEM; *n* = 10 in per group

^b^mPAP, mean pulmonary arterial pressure; Breathing, breathing rate; Systolic, peripheral systolic blood pressure; Diastolic, peripheral diastolic blood pressure; Mean, peripheral mean blood pressure; Heart rate, heart beat rate

^c^One day before the start of NCG feeding

^d^Four weeks after the start of NCG feeding

### Effect of NCG supplementation on NO synthesis parameters

Plasma eNOS, arginine, and NO were significantly lower in the HAPH group compared with the healthy group (*P* = 0.020, *P* = 0.018, *P* = 0.001; respectively, Table 2). However, supplementation with NCG significantly decreased the plasma ammonia concentration (*P* = 0.001), while it increased the plasma eNOS, NO, and arginine concentrations (*P* = 0.001, *P* = 0.002, *P* = 0.001; respectively) in both groups. The state × treatment interaction (*P* = 0.022) significantly affected the plasma ammonia concentration.

**Table 2:** Effects of supplementation with N-carbamylglutamate (NCG 20 g/ d per heifer) on nitric oxide synthesis parameters^a^

Item^b^	Urea, mmol/L	eNOS, U/mL	Arg, μmol/L	NO, μmol/L	Ammonia, μmol/L
Healthy before^c^	4.42	22.26	41.83	23.84	230.66
Healthy after^d^	4.81	29.23	51.42	48.59	20.02
HAPH before	3.93	16.46	50.50	18.40	159.32
HAPH after	4.49	20.19	57.83	33.46	37.09
SEM	0.166	1.275	1.483	2.206	16.457
Main effects					
Status					
Healthy	4.62	25.75	46.63	36.22	125.34
HAPH	4.21	18.33	54.17	25.93	98.21
Treatment					
Before	4.18	19.36	46.17	21.12	194.99
After	4.65	24.71	54.63	41.03	28.56
*P*-value					
State	0.325	0.020	0.018	0.001	0.190
Treatment	0.083	0.001	0.002	0.001	0.001
State×Treatment	0.748	0.100	0.548	0.059	0.022

^a^Healthy = healthy Holstein heifers, HAPH = Holstein heifers with high altitude pulmonary hypertension. Means and pooled SEM; *n* = 10 in per group

^b^Urea, plasma urea; eNOS, endothelial nitric oxide synthase; Arg, arginine; NO, nitric oxide; Ammonia, plasma ammonia

^c^One day before the start of NCG feeding

^d^Four weeks after the start of NCG feeding

## Discussion

The effects of supplementation with NCG in Holstein heifers with HAPH were studied for the first time. Pulmonary hypertension is associated with a disorder of endothelial cell proliferation in humans [33], and reduced expression of NO enzymes in the endothelium of pulmonary arteries with abnormal wall morphology [14]. Furthermore, endothelial NO synthase plays an important role in the maintenance of normal blood pressure and pulmonary vascular wall structure [14]. In the present study, the vascular intima was relatively thinner in heifers in the HAPH group than in the healthy group, which was accompanied by fewer endothelial cells and reduced eNOS expression. It has been reported that the degree of intimal thickening is negatively correlated with the severity of lung disease [34]. In human patients, an inverse correlation was observed between the intimal thickness of blood vessels and impaired NO release [35]. These findings indicate that in heifers with HAPH, the vascular intimal structure was impaired, which in turn decreased the expression of eNOS in the pulmonary arteries.

In the present study, the lower NO production in HAPH heifers could not be attributed to arginine deficiency. This is because *L*-arginine, the substrate for eNOS, is the major determinant of NO synthesis [36], which was higher in heifers in the HAPH group than in the healthy group. A previous study reported similar results, indicating that the impaired NO production is not due to a deficiency in *L*-arginine availability and/or transport [35]. It was further demonstrated that, in the lungs of patients with pulmonary hypertension, and an abnormal wall morphology in the endothelium of pulmonary arteries, eNOS expression was substantially reduced [14].We also found that the expression of eNOS was reduced in HAPH affected heifers, and this reduces the efficiency of plasma arginine utilization. Previous studies have reported that the expression of the *eNOS* gene tends to increase in allantochorion tissue of sow placentas after NCG supplementation [22]. We also found that NCG supplementation increased the plasma concentration of *L*-arginine, eNOS, and NO. We speculated that NCG enhances *L*-arginine synthesis, which may contribute to the restoration of eNOS-coupled activity by reducing the generation of superoxide anions, and increasing NO synthesis [24]. We conclude that a reduction in NO expression in heifers with HAPH was mainly attributed to a decrease in eNOS expression, rather than to a deficiency in plasma *L*-arginine.

Urea cycle disorders are attributed to a deficiency in N-acetylglutamate synthase, which can be successfully treated with NCG and arginine hydrochloride [20]. In Holstein cattle, dietary supplementation with NCG was shown to decrease plasma ammonia and urea concentrations [23]. In the present study, NCG supplementation decreased plasma ammonia concentrations, which suggests that NCG is a potent agent for accelerating ammonia reduction and arginine synthesis.

Endothelial produced NO is a potent vasodilator, and induces vascular smooth muscle relaxation [17]. In the present study, we found that NCG supplementation decreased mPAP in both group. Because mPAP measurement is actually a measure of pulmonary blood flow resistance [34], these results indicate that NCG supplementation reduces pulmonary vascular resistance by generating NO. In cattle, cor pulmonale occurs when pulmonary hypertension, caused by increased pulmonary vascular resistance, which increases the cardiac workload [37]. These results indicate that pulmonary arterial blood pressure can be decreased in Holstein heifers by NCG supplementation, which has beneficial effects in terms of reducing vascular resistance. Increased endogenous NO production, is responsible for the low pulmonary arterial pressure found in high-altitude adapted yaks [12]. Tibetans had > 10-fold higher circulating concentrations of bioactive NO products, which contribute to adaptation to high-altitude hypoxia by increasing blood flow [13]. Actually, augmented NO expression maintain normal levels of oxygen achieve and normal oxygen delivery by promoting blood flow and decreasing vascular resistance in Tibetans [13]. Present study also observed that NCG improved NO synthesis and decreased mPAP. Further, NO induces pulmonary vasodilation not only through increasing the production of cyclic guanosine monophosphate but also indirectly by inhibiting ET-mediated pulmonary vasoconstriction [38]. These findings indicate that supplementation of NCG increase NO expression in heifers ascended to high altitude, which accounts for adaptation to high-altitude hypoxia.

## Conclusions

In conclusion, the findings indicate that eNOS synthesis decreased in Holstein heifers with HAPH, which likely contributed to a deterioration of HAPH. Dietary supplementation with NCG reduced mPAP, peripheral systolic blood pressure, through the restoration of eNOS and endogenous NO synthesis in Holstein heifers with HAPH. Because NCG has a lower rumen degradability compared with arginine, it could be a potential alternative to arginine for attenuation of bovine HAPH, and could also be used to alleviate pulmonary hypertension in human patients.

## Additional file

Additional files 1: **Table S1.** Ingredients and chemical composition of the total mixed ration. (DOCX 14 kb)

## Abbreviations

eNOS: Endothelial nitric oxide synthase
ET-1: Endothelin-1
GAPDH: Glyceraldehyde 3-phosphate dehydrogenase
HAPH: High-altitude pulmonary hypertension
mPAP: Mean pulmonary hypertension
NCG: N-carbamylglutamate
NO: Nitric oxide
RT-PCR: Reverse-transcription polymerase chain reaction
TMR: Total mixed ration
VVG: Verhoeff–Van Gieson staining
α-SMA: Alpha smooth muscle actin
