# Supplementary material for: N-carbamylglutamate restores nitric oxide synthesis and attenuates high altitude-induced pulmonary hypertension in Holstein heifers ascended to high altitude
Source: J Anim Sci Biotechnol. 2018 Sep 3;9:63. doi: 10.1186/s40104-018-0277-6 (PMC6120069; doi:10.1186/s40104-018-0277-6)
Supplement: Supplementary file 1 — Table S1. Ingredients and chemical composition of the total mixed ration. (DOCX 14 kb) [file 40104_2018_277_MOESM1_ESM.docx]

**Additional file**

**Table S1. Ingredients and chemical composition of the total mixed ration.**

| Composition | Amount (%) |
| --- | --- |
| Ingredient/diets (% of dry matter) |  |
| Corn silage | 36.40 |
| Alfalfa hay | 24.36 |
| Oat hay | 9.56 |
| Corn grain | 14.91 |
| Soybean meal | 4.47 |
| Rapeseed meal | 2.09 |
| Cotton seed meal | 1.79 |
| Wheat bran | 1.82 |
| Cottonseed protein | 1.49 |
| Jujube powder | 1.04 |
| Premix^a^ | 0.30 |
| Limestone | 0.50 |
| Dicalcium phosphate | 0.42 |
| Sodium bicarbonate | 0.75 |
| Salt | 0.24 |
| Total | 100.00 |
| Chemical composition^b^ (% dry matter) |  |
| Dry matter | 50 |
| Crude protein | 13.49 |
| NE_L,_ MJ/kg^c^ | 6.02 |
| NFC^d^ | 42.50 |
| Acid detergent fiber | 22.96 |
| Neutral detergent fiber | 34.11 |
| Ether extract | 3.50 |
| Ash | 6.40 |
| Calcium | 0.73 |
| Total phosphorus | 0.47 |
| Forage: concentrate | 70:30 |

^a^One kilogram of complete diet (dry matter basis) contained the following mineral and vitamin premix: 1,000,000 IU vitamin A; 65,000 IU vitamin D; 5,000 IU vitamin E; 2,000 mg Fe; 2,550 mg Mn; 5,500 mg Zn; 1,750 mg Cu; 70 mg I; 40 mg Co; 75 mg Se

^b^Measured from laboratory analysis of the total mixed ration

^c^Calculated using net energy of lactation values of feedstuffs from NRC (2001)

^d^Nonfiber carbohydrates 100– (NDF% + CP% + Ether extract% + Ash%)
